# Investigation of the Antibacterial Activity and Efflux Pump Inhibitory Effect of *Cycas thouarsii* R.Br. Extract against *Klebsiella pneumoniae* Clinical Isolates

**DOI:** 10.3390/ph14080756

**Published:** 2021-08-01

**Authors:** Walaa A. Negm, Mona El-Aasr, Amal Abo Kamer, Engy Elekhnawy

**Affiliations:** 1 Pharmacognosy Department, Faculty of Pharmacy, Tanta University, Tanta 31111, Egypt; moelaasar@pharm.tanta.edu.eg; 2 Pharmaceutical Microbiology Department, Faculty of Pharmacy, Tanta University, Tanta 31111, Egypt; amalabokamer@pharm.tanta.edu.eg

**Keywords:** biflavonoids, efflux, membrane permeability, natural products, SEM, 5,7,7″,4″-tetra-*O*-methyl-hinokiflavone

## Abstract

The vast spread of multidrug-resistant bacteria has encouraged researchers to explore new antimicrobial compounds. This study aimed to investigate the phytochemistry and antibacterial activity of *Cycas thouarsii* R.Br. leaves extract against *Klebsiella pneumoniae* clinical isolates. The minimum inhibitory concentration (MIC) values of *C. thouarsii* extract ranged from 4 to 32 µg/mL. The impact of the treatment of the isolates with sub-inhibitory concentrations of *C. thouarsii* extract was investigated on the bacterial growth, membrane integrity, inner and outer membrane permeability, membrane depolarization, and bacterial morphology using a scanning electron microscope (SEM) and on the efflux activity using qRT-PCR. Interestingly, most *K. pneumoniae* isolates treated with *C. thouarsii* extract showed growth inhibition—a decrease in membrane integrity. In addition, we observed various morphological changes, a significant increase in inner and outer membrane permeability, a non-significant change in membrane depolarization, and a decrease in efflux activity after treatment. The phytochemical investigation of *C. thouarsii* extract revealed the isolation of one new biflavonoid, 5,7,7″,4‴-tetra-*O*-methyl-hinokiflavone (**3**), and five known compounds, stigmasterol (**1**), naringenin (**2**), 2,3-dihydrobilobetin (**4**), 4′,4‴-*O-*dimethyl amentoflavone (**5**), and hinokiflavone (**6**), for the first time. Moreover, the pure compounds′ MICs′ ranged from 0.25 to 2 µg/mL. Thus, *C. thouarsii* could be a potential source for new antimicrobials.

## 1. Introduction

*Klebsiella pneumoniae* is a Gram-negative, rod-shaped bacterium that belongs to the family *Enterobacteriaceae* [1]. It has emerged as an important opportunistic pathogen, causing nosocomial infections, especially in urinary, respiratory tracts, and blood [2]. Recently, multidrug resistance is highly disseminated among *Klebsiella pneumoniae* isolates causing serious problems at the clinical sites due to the decreased therapeutic options available to treat such resistant bacteria [3]. *K. pneumoniae* isolates could acquire different mechanisms that lead to antibiotic resistance to many antibiotics [4]. This concern has resulted in high mortality rates and extended hospitalization periods in patients infected with this pathogen [5]. Consequently, studies with alternative approaches, for example, herbal medicines, are emerging as a promising trend to combat such resistant isolates.

The *Cycas thouarsii* R.Br.′s family, Cycadaceae, is the only African representative of the *Cycas*, the Cycadaceae′s sole genus. It is a widespread fast-growing species found mainly on Madagascar′s east coast [6]. Recently, *Cycas thouarsii* was reported to display cytotoxic, antioxidant, and antimicrobial activities [7]. The *Cycas* genus contains a wide variety of flavonoids and biflavonoids as well as their glycosides, which are responsible for the activity of their plants [8,9,10]. Through the continuous search for new antibacterial agents from different plants, we aimed in this study to examine the antibacterial activity of *Cycas thouarsii* R.Br. leaves extract against *Klebsiella pneumoniae* clinical isolates. In addition, phytochemical investigation, the isolation of pure compounds, the antibacterial effect of the isolated compounds, and the effect on the bacterial membrane properties and efflux activity were investigated.

## 2. Results

### 2.1. Phytochemical Investigation

#### 2.1.1. Spectroscopic Data

**Compound** (**1**): White crystal; m.p. (161–165 °C), IR (KBr) *v_ma__x_*: 3449, 2930, 2861, 1644, 1462, 1377, 882, 830. ^1^H NMR [CDCl_3_, 500 MHz] δ: 3.52 (1H, m, H-3), 5.35 (1H, t, H-6), 0.67 (3H, s,H-18) 0.92 (3H, d, *J* = 6.0 Hz, H-19), 5.00 (1H, m, H-20), 5.15 (1H, m, H-21), 0.84 (3H, t), 0.82 (3H, d, *J* = 6.0 Hz, H-26), 0.81 (3H, d, *J* = 6.0 Hz, H-27), 0.69 (3H, s, H-28), 1.00 (3H, s, H-29). ^13^C NMR [CDCl_3_, 125 MHz] δ: 37.22 (C-1), 30.94 (C-2), 71.80 (C-3), 42.20 (C-4), 140.73 (C-5), 121.72 (C-6), 31.63 (C-7), 31.87 (C-8), 50.09 (C-9), 36.48 (C-10), 21.06 (C-11), 39.65 (C-12), 42.28 (C-13), 56.7 (C-14), 24.29 (C-15), 28.24 (C-16), 56.01 (C-17), 39.74 (C-18), 21.05 (C-19), 138.32 (C-20), 129.24 (C-21), 45.80 (C-22), 26.01 (C-23), 11.84 (C-24), 29.09 (C-25), 19.81 (C-26), 19.38 (C-27), 19.0 (C-28), 12.03 (C-29). EI-MS *m/z* 412.63 (M^+^), with a molecular formula of C_29_H_48_O.

**Compound** (**2**): Slight yellow powder, IR (KBr) *v_max_:* 3957, 3780, 3290, 3119, 2918, 2831, 2709, 1634 cm^−1^. ^1^H NMR [DMSO-*d*_6_, 500 MHz] δ_H_: 5.42 (1H, dd, *J* = 12.5, 3.5 Hz, H-2), 3.30 (1H, dd, *J* = 17.0, 12.5 Hz, H-3a), 2.69 (1H, dd, *J* = 17.0, 3.5 Hz, H-3b), 5.88 (2H, d, *J* = 2.0 Hz, H-6, 8), 7.31 (2H, d, *J* = 8.0 Hz, H-2′, 6′), 6.82 (2H, d, *J* = 8.0 Hz, H-3′, 5′), 12.15 (OH at 5). ^13^C NMR [DMSO-*d_6_*, 125 MHz] δ_C_: 79.04 (C-2), 42.16 (C-3), 196.38 (C-4), 164.03 (C5), 95.86 (C-6), 166.92 (C-7), 94.80 (C-8), 163.45 (C-9), 101.94 (C-10), 129.66 (C-1′), 127.63 (C-2′, 6′), 114.91 (C-3′, 5′), 157.56 (C-4′) ESI-MS *m/z* 271.223 [M-H]^−^, with a molecular formula of C_15_H_12_O_5_.

**Compound** (**3**): Yellow amorphous powder, [α] ^25^_D_ = −27.38° (CH_3_OH, c = 0.5), IR (KBr disc) *v*_max_: 3451, 3078, 2939, 2841, 1981, 1657, 1606, 1434, 1373, 1339, 1262, 1060, 1028, 835 cm^−1^. ^1^H NMR [CDCl_3_, 500 MHz] δ_H_: 6.66 (1H, s, H-3), 6.36 (1H, d, *J* = 2.5 Hz, H-6), 6.44 (1H, d, *J* = 2.5 Hz, H-8), 6.61 (1H, s, H-3″), 6.54 (1H, s, H-8″), 7.88 (2H, d, *J* = 8.5 Hz, H-2′, 6′), 7.18 (2H, d, *J* = 8.5 Hz, H-3′, 5′), 7.48 (2H, d, *J* = 8.5 Hz, H-2‴, 6‴), 6.84 (2H, d, *J* = 8.5 Hz, H-3‴, 5‴), 13.12 (OH at 5″), 3.79, 3.81, 3.84, 3.86 (OCH_3_ at 5, 7, 7″, and 4‴). ^13^C NMR [CDCl_3_, 125 MHz] δ_C_: 164.04 (C-2), 105.06 (C-3), 182.81 (C-4), 162.11 (C5), 98.15 (C-6), 162.52 (C-7), 92.62 (C-8), 157.70 (C-9), 104.57 (C-10), 131.07 (C-1′), 127.73 (C-2′), 111.19 (C-3′), 162.36 (C-4′), 111.19 (C-5′), 127.73 (C-6′), 165.44 (C-2″), 103.56 (C-3″), 182.37 (C-4″), 155.10 (C-5″), 123.34 (C-6″), 157.70 (C-7″), 94.43 (C-8″), 154.09 (C-9″), 105.52 (C-10″), 127.98 (C-1‴), 128.00 (C-2‴, 6‴), 114.48 (C-3‴, 5‴), 160.65 (C-4‴), 56.33, 55.94, 55.79, 55.48 (OCH_3_ at C-5, 7, 7”, and 4‴); Data assigned by COSY and HMBC. ESI-MS *m/z* 593.3633 [M-H]^−^, with a molecular formula of C_34_H_26_O_10_.

**Compound** (**4**): Yellowish white amorphous powder; IR (KBr) *v_max_*: 3418, 2958, 2928, 1729, 1655, 1605, 874. ^1^H NMR [DMSO-*d*_6_, 500 MHz] δ_H_: 5.36–5.63 (1H, m, H-2), 3.20–3.32 (1H, m, H-3a), 2.67–2.92 (1H, m, H-3b), 5.81 (1H, m, H-6), 5.91 (1H, m, H-8), 7.46–7.52 (2H, m, H-2′, 6′), 7.40 (1H, d, *J* = 8.0 Hz, H-5′), 6.51 (1H, s, H-3″), 6.25 (1H, s, H-6″), 7.41 (2H, dd, *J* = 8.0, 3.0 Hz, H-2‴, 6‴), 6.65–6.70 (2H, m, H-3‴, 5‴), 3.65 (OCH_3_ at 4′). ^13^C NMR [DMSO-*d*_6_, 125 MHz] δ_C_: 79.08 (C-2), 43.16 (C-3), 192.23 (C-4), 164.33 (C5), 95.70 (C-6), 166.52 (C-7), 94.87 (C-8), 163.45 (C-9), 101.73 (C-10), 130.66 (C-1′), 131.33 (C-2′), 121.19 (C-3′), 158.26 (C-4′), 110.70 (C-5′), 127.97 (C-6′), 164.08 (C-2″), 102.63 (C-3″), 184.23 (C-4″), 164.33 (C5″), 98.36 (C-6″), 162.52 (C-7″), 104.57 (C-8″), 155.45 (C-9″), 104.50 (C-10″), 122.66 (C-1‴), 127.92 (C-2‴, 6‴), 115.49 (C-3‴, 5‴), 161.31 (C-4‴), 54.78 (OCH_3_ at C-4′). ESI-MS *m/z* 553.269 [M-H]^−^, with a molecular formula of C_31_H_22_O_10_.

**Compound** (**5**): Yellow amorphous powder; IR (KBr) *v_max_*: 3396, 2966, 2925, 2859, 2318, 1656, 1602, 1440, 1379, 1339, 1256, 1093, 882, 836. ^1^H NMR [DMSO-*d*_6_, 500 MHz] δ_H_: 6.92 (1H, s, H-3), 6.18 (1H, d, *J* = 2.0 Hz, H-6), 6.47 (1H, d, *J* = 2.0 Hz, H-8), 8.06 (1H, d, *J* = 2.0 Hz, H-2′), 7.31 (1H, d, *J* = 9.0 Hz, H-5′), 8.18 (1H,dd, *J* = 2.0, 9.0 Hz, H-6′), 6.78 (1H, s, H-3″), 6.38 (1H, s, H-6″), 7.50 (2H, d, *J* = 8.5 Hz, H-2‴, 6‴), 6.71 (2H, d, *J* = 8.5 Hz, H-3‴, 5‴), 10.28, 10.84 OH at C7, 7″), 3.77, 3.79 (OCH_3_ at 4′, 4‴). ^13^C NMR [DMSO-*d*_6_, 125 MHz] δ_C_: 163.61 (C-2), 103.60 (C-3), 182.11 (C-4), 161.46 (C5), 98.59 (C-6), 163.40 (C-7), 94.15 (C-8), 157.45 (C-9), 103.63 (C-10), 121.66 (C-1′), 128.30 (C-2′), 121.27 (C-3′), 160.64 (C-4′), 111.71 (C-5′), 130.61 (C-6′), 164.25 (C-2″), 103.36 (C-3″), 181.82 (C-4″), 160.48 (C5″), 98.94 (C-6″), 161.81 (C-7″), 104.60 (C-8″), 154.32 (C-9″), 103.70 (C-10″), 122.48 (C-1‴), 128.17 (C-2‴), 115.66 (C-3‴), 161.10 (C-4‴), 115.83 (C-5″), 128.08 (C-6‴), 56.03, 55.93 (OCH_3_ at C-4′, 4‴). ESI-MS *m/z* 565.362 [M-H]^−^, with a molecular formula of C_32_H_22_O_10_.

**Compound** (**6**): Yellow amorphous powder IR (KBr disc) *v*_max_: 3450, 2927, 2858, 2358, 1643, 1430, 1364, 1287, 835 cm^−1^. ^1^H NMR [DMSO-*d*_6_, 500 MHz] δ_H_: 6.83 (1H, s, H-3), 6.17 (1H, d, *J* = 2.0 Hz, H-6), 6.43 (1H, d, *J* = 2.0 Hz, H-8), 6.77(1H, s, H-3″), 6.34 (1H, s, H-8″), 8.04 (2H, d, *J* = 8.5 Hz, H-2′, 6′), 7.08 (2H, d, *J* = 8.5 Hz, H-3′, 5′), 7.97 (2H, d, *J* = 8.5 Hz, H-2‴, 6‴), 6.86 (2H, d, *J* = 8.5 Hz, H-3‴, 5‴). ^13^C NMR [DMSO-*d*_6_, 125 MHz] δ_C_: 163.95 (C-2), 103.67 (C-3), 181.33 (C-4), 161.43 (C5), 98.82 (C-6), 163.52 (C-7), 94.03 (C-8), 157.10 (C-9), 103.52 (C-10), 124.27 (C-1′), 127.75 (C-2′), 115.19 (C-3′), 162.52 (C-4′), 115.19(C-5′), 127.75 (C-6′), 164.09 (C-2″), 103.26 (C-3″), 182.03 (C-4″), 155.11 (C-5″), 124.34 (C-6″), 157.33 (C-7″), 94.61 (C-8″), 153.59 (C-9″), 104.52 (C-10″), 121.51 (C-1‴), 128.20 (C-2‴, 6‴), 116.12 (C-3‴, 5‴), 160.51 (C-4‴). ESI-MS *m/z* 537.082 [M-H]^−^, with a molecular formula of C_30_H_18_O_10_.

#### 2.1.2. Structure Elucidation of the Isolated New Compound (**3**)

Dried leaves of *Cycas thouarsii* were extracted with methanol by cold maceration and then successively partitioned with *n*-hexane, dichloromethane, ethyl acetate, and *n*-butanol, respectively. The dichloromethane fraction residue (DCM) was subjected to different columns chromatography to yield six compounds. Their chemical structures were identified by spectroscopic analysis (1D, 2D NMR, and ESI/MS) and comparison with the reported data.

**Figure 1:**
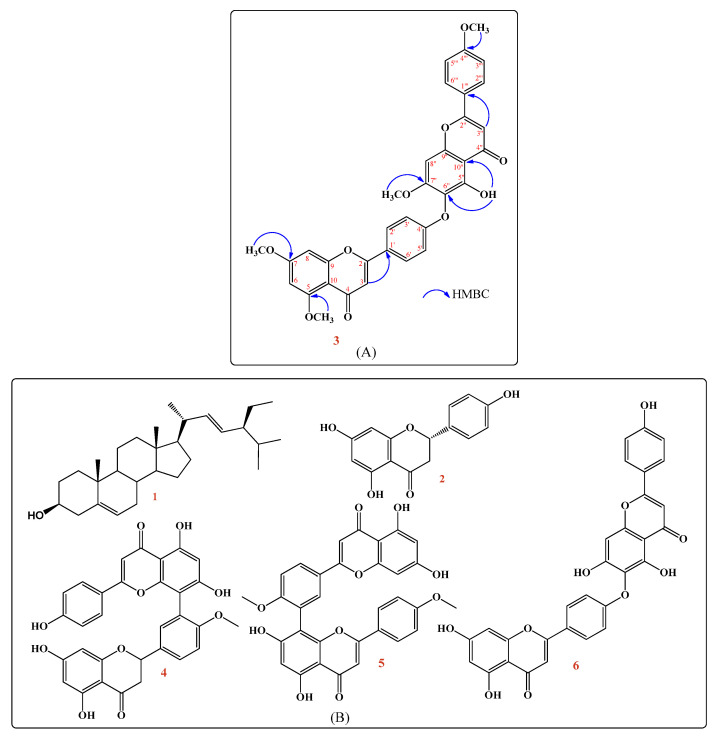
(**A**): The chemical structure of the new compound (**3**) showing HMBC correlations. (**B**): The chemical structures of isolated pure compounds.

Compound (**3**) is a new biflavonoid, and the ^1^H-NMR of **3** established the presence of only one hydroxyl group that resonated at δ 13.12, indicating the presence of one chelated hydroxyl at the 5″ position. The ^1^H-NMR spectrum also showed two sets of the AA′BB′ systems as indicated by δ 7.48 (2H, d, *J =* 8.5 Hz) for H-2‴, 6‴, δ 6.84 (2H, d, *J =* 8.5 Hz) for H-3‴, 5‴ and 7.88 (2H, d, *J =* 8.5 Hz) for H-2′, 6′, and δ 7.18 (2H, d, *J =* 8.5 Hz) for H-3′, 5′. The presence of two *meta*-coupled doublets (*J* = 2.5 Hz) each due to the one proton in the upfield aromatic region at δ 6.36 and 6.44 was ascribed to the H-6 and H-8 protons, respectively. Moreover, the three-singlet signals at δ 6.54, 6.61, and 6.66 were assigned to H-8″, H-3″, and H-3, suggesting the biflavonoid linkage between 4′-OH and 6″. The ^1^H-NMR spectrum revealed a hinokiflavone pattern (a diaryl ether-type biflavonoid) with four aromatic methoxy signals at δ 3.79, 3.81, 3.84, and 3.86, suggesting tetra-*O*-methyl derivatives. The ^13^C-NMR spectrum further confirmed the biflavonoid structure from the presence of 30 carbons in addition to four methoxy signals at δ 56.33, 55.94, 55.79, and 55.48 ppm. The ^13^C-NMR spectrum showed the presence of one signal at δ 92.62 and two signals at δ 98.15 and 95.43 assigned to C-8, C-6, and C-8″, respectively. This implies that C-6″ is involved in the interflavonoid linkage, which also confirmed the hinokiflavone pattern [11]. Hinokiflavone *O*-methylation affects the shift of *ortho*- and *para*-atoms, as the ^13^C-NMR spectrum revealed a downfield shift in C-10, an upfield shift in C-8 indicating 7-*O*-methoxylation, a downfield shift in C-10″, and an upfield shift in C-8″ indicating 7″-*O*-methoxylation. While there was an upfield shift in C-3‴, 5‴, there was a downfield shift in C-1‴, indicating 4‴-*O*-methoxylation compared with hinokiflavone (**6**) [12]. The tetra-*O*-methyl-hinokiflavone structure confirmed with HMBC correlations from OCH_3_-7 at δ 3.86 to C-7 at δ 162.52, from OCH_3_-7″ at δ 3.84 to C-7″ at δ 157.70, from OCH_3_-4‴ at δ 3.81 to C-4‴ at δ 160.65, from OCH_3_-5 at δ 3.79 to C-5 at δ 162.11, and from OH-5″ at δ 13.12 to C-6″ at δ 123.34 and C-10″ at δ 105.52 [13]. All correlations are displayed in Figure 1A. The ESI-MS spectrum of this compound showed ion at *m/z* 593.3633 [M-H]^−^ which matches with the determined structure. Only mono-methoxy (isocryptomerin and cryptomerin B) and di-methoxy (chamaecyparin) derivatives of hinokiflavone were isolated from plants [12,14]. Thus, this compound was identified as 5,7,7″,4‴-tetra-*O*-methyl-hinokiflavone (**3**) and considered a new compound isolated for the first time from all plants. Other isolated known compounds were identified as stigmasterol (**1**) [8], naringenin (**2**) [9], 2,3-dihydrobilobetin (**4**) [10], 4′,4‴-*O*-dimethyl amentoflavone or isoginkgetin (**5**) [10], hinokiflavone (**6**) [12] for the first time from *C. thouarsii* R.Br. leaves extract. The chemical structures of the isolated compounds are displayed in Figure 1.

### 2.2. Biological Investigation

*C. thouarsii* extract exhibited an antibacterial effect on the tested *K. pneumoniae* isolates using the agar well diffusion method. The minimum inhibitory concentration (MIC) values for the *C. thouarsii* extract were determined by the broth microdilution method, and they ranged from 4 to 32 µg/mL.

#### 2.2.1. Bacterial Growth Curve

**Figure 2:**
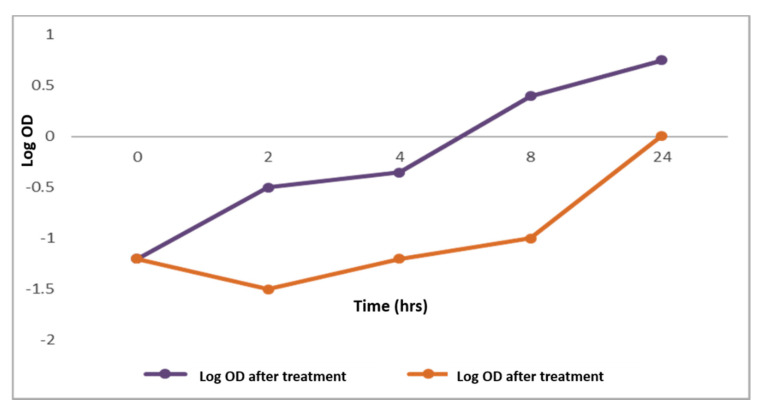
Growth curve of *K. pneumoniae* representative isolates before and after treatment with *C. thouarsii* extract (16 µg/mL).

*C. thouarsii* extract (at concentrations ranging from 2 to 16 µg/mL) inhibited the growth cycle curve and decreased the growth in 73.5% of the tested isolates using a spectrophotometric method [15]. A representative example is shown in Figure 2.

#### 2.2.2. Integrity of Cell Membranes

**Figure 3:**
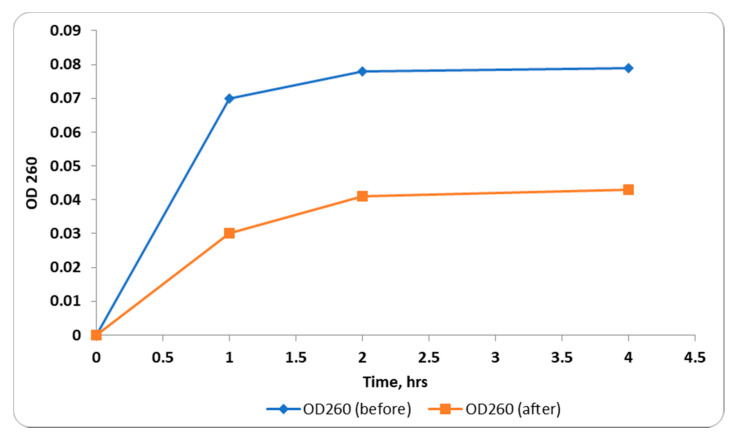
Release of 260 nm absorbing material from representative *K. pneumoniae* isolates before and after treatment with *C. thouarsii* extract (16 µg/mL).

The integrity of the tested bacterial membranes was investigated before and after treatment with *C. thouarsii* extract (at concentrations ranging from 2 to 16 µg/mL) via monitoring the release of the material, absorbing at 260 nm, from the tested bacteria [16]. A significant decrease in membrane integrity (Figure 3) was detected in 73.5% of the treated cells.

#### 2.2.3. Inner Membrane Permeability Assay

**Figure 4:**
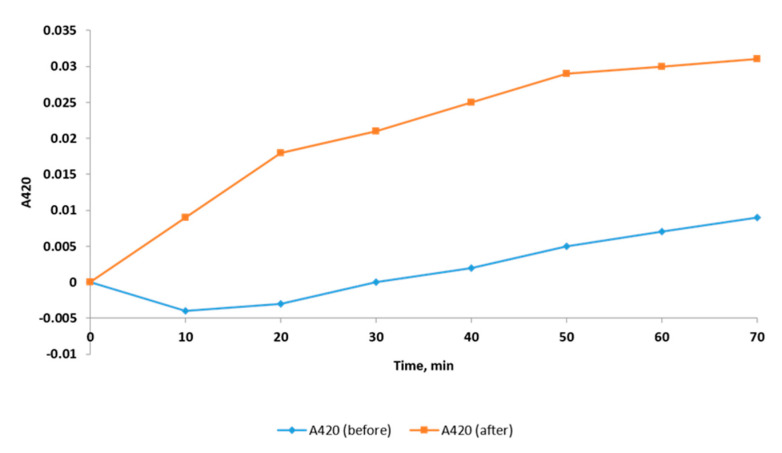
Representative example of change in the inner membrane permeability of *K. pneumoniae* isolates before and after treatment with *C. thouarsii* extract (16 µg/mL).

When the inner membrane of the tested bacterial cells is permeable, *ortho*-nitrophenyl-*β*-galactopyranoside (ONPG) enters the cytoplasm and is broken down by the *β*-galactosidase enzyme to *ortho*-nitrophenol (ONP, yellow color). The production of ONP was monitored by determination of the increase in the absorbance (at an optical density (OD) of 420) with time [17] as demonstrated in Figure 4. A significant increase in the inner membrane permeability was detected after treatment with *C. thouarsii* extract (at concentrations ranging from 2 to 16 µg/mL) in 67.6% of isolates.

#### 2.2.4. Outer Membrane Permeability

**Figure 5:**
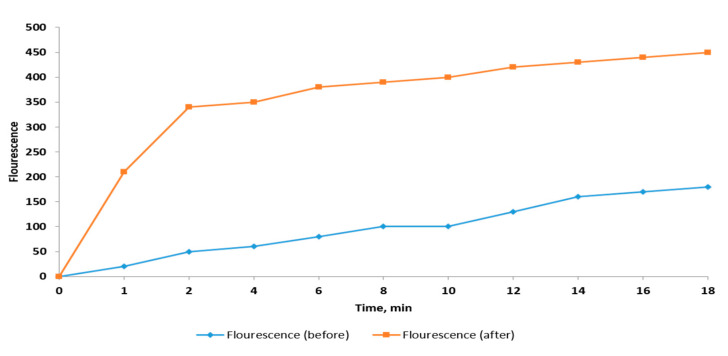
Representative example of the change in the outer membrane permeability of *K. pneumoniae* isolates before and after treatment with *C. thouarsii* extract (16 µg/mL).

Outer membrane permeability was measured by recording the fluorescence of the hydrophobic agent 1-*N*-phenylnaphthylamine (NPN) using a spectrofluorophotometer [18]. The NPN fluorescence is detectable in hydrophobic environments such as the hydrophobic region of the cell membrane. As shown in Figure 5, a significant increase in the fluorescence of NPN, hence, the outer membrane permeability, was found in 58.8% of the treated isolates (concentrations of *C. thouarsii* extract ranged from 2 to 16 µg/mL).

#### 2.2.5. Membrane Depolarization Assay

**Figure 6:**
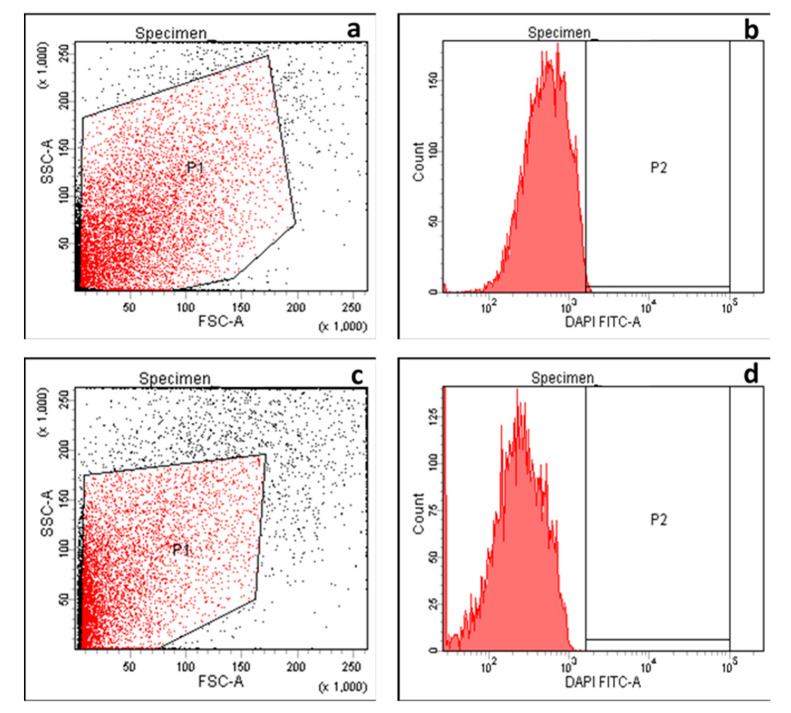
Flow cytometric dot plot and histogram showing fluorescence detected by a FACSverse flow cytometer in a representative *K. pneumoniae* isolate before treatment (dot plot (**a**) and histogram (**b**)) and after treatment (dot plot (**c**) and histogram (**d**)) with *C. thouarsii* extract (16 µg/mL).

Flow cytometry was utilized to obtain a quantitative prospect regarding the impact of *C. thouarsii* extract on membrane depolarization of the tested bacterial isolates. Flow cytometric measurements were conducted on the tested cells after staining with bis-(1,3-dibutyl barbituric acid) trimethineoxonol [DiBAC4(3)], an anionic membrane potential-sensitive fluorescent agent that can access the depolarized cells where it binds to the membrane or intracellular proteins exhibiting enhanced fluorescence [17,19]. A non-significant change in the membrane depolarization after treatment with *C. thouarsii* extract (at concentrations ranging from 2 to 16 µg/mL). A representative example of the flow cytometric dot plots and histograms before and after treatment are revealed in Figure 6.

#### 2.2.6. Scanning Electron Microscope (SEM) Examination

**Figure 7:**
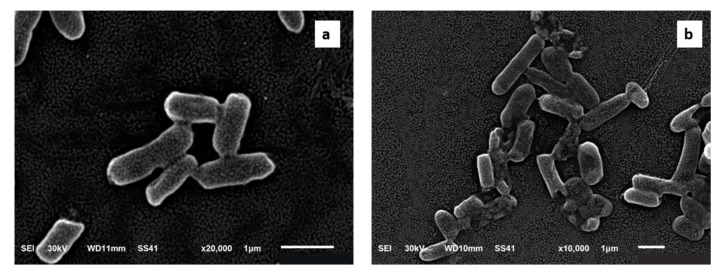
Scanning electron microscope image of a representative *K. pneumoniae* isolates (**a**) before and (**b**) after treatment with *C. thouarsii* extract (16 µg/mL).

The impact of *C. thouarsii* extract (at concentrations ranging from 2 to 16 µg/mL) on the bacterial morphology was studied using SEM [20]. As shown in Figure 7, many morphological alterations were revealed involving wrinkling of the cell surface. In addition, disruption of the cell wall was noticed represented by the appearance of certain holes and cracks or even total cell lysis. Moreover, certain clusters of lysed cells were detected.

#### 2.2.7. Detection of Efflux

In the present study, *K. pneumoniae* isolates showed a decrease in the efflux activity after treatment with *C. thouarsii* extract (16 µg/mL) as shown in Table 1. Isolates lacking efflux activity fluoresced at an ethidium bromide (EtBr) concentration of 0.5 mg/L [21].

#### 2.2.8. Quantitative RT-PCR

To determine the impact of *C. thouarsii* extract on the efflux pump systems, the expression of four efflux pump genes in nine *K. pneumoniae* isolates before and after treatment with *C. thouarsii* extract (2 to 16 µg/mL) was inspected, and the results are shown in Table 2. Melting curve analysis was used to evaluate primer-dimers and other artifacts.

#### 2.2.9. Antimicrobial Activity of Different Fractions and Isolated Pure Compounds

Different fractions of *C. thouarsii* exhibited antibacterial activity against *K. pneumoniae* clinical isolates with MIC values ranging from 16 to 512 µg/mL. The DCM fraction had the highest activity (16–32 µg/mL), followed by ethyl acetate (32–128 µg/mL), then *n*-butanol fraction (128–512 µg/mL), while the *n*-hexane fraction exhibited the lowest antibacterial activity (256–1024 µg/mL). The MIC values of the pure isolated compounds ranged from 0.25 to 2 µg/mL. Among the isolated compounds, hinokiflavone (**6**) presented the highest activity (0.25–0.5 µg/mL), followed by naringenin (**2**) (0.5–1 µg/mL), 2,3-dihydrobilobetin (**4**) (0.5–2 µg/mL), 4′,4‴-*O*-dimethyl amentoflavone or isoginkgetin (**5**) (1–2 µg/mL), while 5,7,7”,4”′-tetra-*O*-methyl-hinokiflavone (**3**) and stigmasterol (**1**) presented moderate activity (1.5–2 µg/mL).

## 3. Discussion

Recently, the search for novel antibacterial agents has become a vital aim owing to the increasing levels of antibiotic resistance amongst pathogenic bacteria [22]. A promising trend in this aspect involves a focus on making use of medicinal plants that have many advantages including widely available resources, low or no side effects, low cost, in addition to having many antimicrobial properties [23]. To our knowledge, this is the first time the antibacterial activity of *C. thouarsii* extract against clinical isolates of *K. pneumoniae* has been investigated.

*K. pneumoniae* is an opportunistic pathogen that infects immunocompromised individuals causing a wide range of infections [24]. In the current work, *C. thouarsii* methanol extract exhibited antibacterial activity against *K. pneumoniae* clinical isolates with MIC values ranging from 4 to 32 µg/mL.

For more comprehension of the impact of *C. thouarsii* extract on *K. pneumoniae*, a bacterial growth curve was constructed by plotting log OD_620_ versus time before and after treatment. Suppression of the bacterial growth was found in 73.5% of the tested isolates. For clarification of how *C. thouarsii* extract was able to inhibit bacterial proliferation, the integrity of the cell membrane was tested. A significant increase (*p* < 0.05) in the release of nucleotides (DNA and RNA) from the cells was detected in 73.5% of the tested isolates which could be explained by induction of membrane disruption by *C. thouarsii* extract.

This was confirmed by testing the inner and outer membrane permeability. A significant increase in the inner and outer membrane permeability was detected after treatment with *C. thouarsii* extract in 67.6% and 58.8% of the isolates, respectively. Despite the impact of *C. thouarsii* extract on membrane properties, including the integrity and the inner and outer membrane permeability, it had a non-significant effect on membrane depolarization measured using flow cytometry. The bacterial membrane is a target for many antimicrobials, and some researchers have reported that certain plant extracts could influence membrane integrity [25] and membrane permeability [26].

To investigate the effect of *C. thouarsii* extract on bacterial morphology, we examined *K. pneumoniae* isolates using SEM before and after treatment. Some morphological changes were detected after treatment, indicating that the extract could completely collapse and lyse the bacterial cells. Latha et al. [27] reported morphological changes induced by certain plant extracts in *Pseudomonas aeruginosa* isolates.

Efflux pump-mediated resistance to antimicrobial agents has constricted the therapeutic options against many bacterial infections. Efflux pumps could reduce the concentration of antibiotics inside the bacterial cells, thus decreasing the antibiotic effect on bacteria [25]. Unfortunately, many bacterial isolates develop resistance to many antibiotics via an efflux pump system [28]. Therefore, we tested the effect of *C. thouarsii* extract on the efflux system in the tested *K. pneumoniae* isolates using fluorometric analysis of the efflux of EtBr (an efflux pump substrate) [29]. Noteworthy is the percentage of the isolates that showed fluorometric efflux of EtBr, which decreased from 70.6% (24 out of 34 isolates) to 32.3% (11 out of 34 isolates) after treatment with *C. thouarsii* extract. This was further elucidated using qRT-PCR to detect the impact of *C. thouarsii* extract treatment on the expression of the genes encoding efflux pump. Our results showed that treatment with *C. thouarsii* extract decreased the expression of *nor*E, *acr*B, *mdf*A, and *yih*V efflux pump genes in 33.3%, 55.5%, 55.5%, and 44.4% of the selected *K. pneumoniae* isolates, respectively.

## 4. Materials and Methods

### 4.1. Preparation of Plant Extract and Isolation of Pure Compounds

Leaves from the *Cycas thouarsii* R.Br. family Cycadaceae were collected from El Abd Garden in Giza city on 14 January 2017. The identity of the plant was kindly confirmed by Esraa Ammar, Plant Ecology lecturer, Botany Department, Faculty of Science, Tanta University, and researcher, Rabea Sharawy, Agronomist and palm researcher. A voucher specimen (PGG-004) is kept at the Pharmacognosy Department, Faculty of Pharmacy, Tanta University.

The plant powdered material (1750 g) was extracted with methanol by percolation (4 × 5 L). The extract was concentrated under reduced pressure to afford a residue (208 g). Total methanol residue (75 g) was resuspended in MeOH: H_2_O (1:1) then successively partitioned with *n*-hexane, dichloromethane, ethyl acetate, and finally *n*-butanol saturated with water to yield 18.28, 23.74, 3.82, and 14.59 g residues, respectively.

Dichloromethane fraction (10 g) of *C. thouarsii* was subjected to a silica gel column chromatography (ϕ 3.5 × 80 cm, 200 g silica, fraction collected 50 mL) using the gradient elution method, starting with CH_2_Cl_2_, and increasing the polarity using MeOH to afford four fractions (fr. A1–A4). Fr. A1 (557 mg, CH_2_Cl_2_–MeOH; 96:4 eluate), Fr. A2 (1042 mg, CH_2_Cl_2_–MeOH; 94:6 eluate), Fr. A3 (900 mg, CH_2_Cl_2_–MeOH; 92:8 eluate), and Fr. A4 (617 mg, CH_2_Cl_2_–MeOH; 84:16 eluate). Fr. A1 (557 mg, CH_2_Cl_2_–MeOH; 96:4 eluate) was subjected to isocratic column chromatography using silica gel, eluted with 100% CHCl_3_. Subfractions (19–23) were collected and recrystallized with methanol to yield white crystals of compound **1** (13 mg). Fr. A2 (1042 mg, CH_2_Cl_2_–MeOH; 94:6 eluate) was subjected to column chromatography on silica gel and eluted with CHCl_3_ and increasing polarity using MeOH to afford 6 fractions. Fr. 2 (231 mg) eluted with (CHCl_3_–MeOH; 92:8) was chromatographed on a silica gel column and eluted with *n*-hexane and increasing polarity using EtOAc (subfractions 8–12, *n*-hexane–EtOAc; 10:90) were collected to yield compound **2** (7 mg). Fr. 5 (300 mg) were subjected to isocratic column chromatography (CH_2_Cl_2_: MeOH; 99:1) to give yellow powder (53 mg) purified on Sephadex LH-20 eluted with 100% MeOH) to obtain compound **3** (9 mg). Fr. A3 (900 mg, CH_2_Cl_2_–MeOH; 92:8 eluate) was subjected to column chromatography on silica gel and eluted with CH_2_Cl_2_ and increasing polarity using MeOH to give 5 subfractions. Subfraction 2 (82 mg), eluted with (CH_2_Cl_2_–MeOH; 90:10), was purified on Sephadex LH-20 eluted with 100% MeOH to afford compound **4** (10 mg). Subfraction 3 (397 mg), eluted with (CH_2_Cl_2_–MeOH; 88:12), was subjected to silica gel column chromatography eluted with CHCl_3_–EtOAc gradient elution to afford 3 fractions. Fraction 2 (100 mg), eluted with (CHCl_3_–EtOAc; 90:10), was further purified on Sephadex LH-20 eluted with 100% MeOH to give compound **5** (12 mg). Fr. A4 (617 mg, CH_2_Cl_2_–MeOH; 84:16 eluate) was subjected to silica gel column chromatography eluted with CH_2_Cl_2_–MeOH; 90:10), then purified on Sephadex LH-20 eluted with 100% MeOH to afford compound **6** (7 mg) (Scheme S1).

### 4.2. Bacterial Isolates

A total of 34 *K. pneumoniae* isolates were collected from different departments of Tanta University Hospital. The clinical isolates were examined microscopically and were identified using standard biochemical tests according to MacFaddin [30]. *Klebsiella pneumoniae* (ATCC 13883) was utilized as a reference strain.

### 4.3. Chemicals

All chemicals used in the current study were purchased from Sigma–Aldrich (St. Louis, MO, USA), except DiBAC4(3) from Invitrogen (Waltham, MA, USA) and NPN from Himedia (Mumbai, India).

### 4.4. Antibacterial Screening

It was performed by the agar well diffusion method as previously described [31]. Briefly, 100 µL (10^6^ CFU/mL) of bacterial suspension was spread on the surface of the Muller Hilton agar (MHA) plate using a swab. Then, 3 wells 6 mm in diameter were punched off using a sterile cork-borer, and each well was filled with 100 μL (32 µg/mL) of *C. thouarsii* extract using DMSO as a negative control and ciprofloxacin as a positive control. The plates were then incubated at 37 °C for 24 h.

### 4.5. Determination of the MIC Values

The broth microdilution method was utilized for the determination of the MIC value of *C. thouarsii* extract for each tested isolate in Muller–Hinton broth (MHB) (Oxoid, Basingstoke, UK) [32]. Equal volumes (100 µL) of bacteria and serial twofold dilutions of *C. thouarsii* extract (from 32 to 500 µg/mL) in MHB were mixed in the wells of a 96-well microtitration plate. Each plate had a positive control (untreated bacteria) and negative control (MHB only). After determination of the MICs of *C. thouarsii* extract for each isolate, all the following experiments were carried out before and after treatment of the tested isolates with a sub-inhibitory concentration (0.5 MIC) of *C. thouarsii* extract.

### 4.6. Bacterial Growth Curve

The impact of *C. thouarsii* extract on the growth of *K. pneumoniae* isolates was examined using a spectrophotometric method [15]. In brief, bacterial cultures were incubated in a shaking incubator (New Brunswich, NJ, USA) at 37 °C for 24 h at 90 rpm. Then, a 4 mL suspension was taken from each flask at time intervals of 0, 2, 4, 6, 8, and 24 h. The OD values were determined at 620 nm with an 1800 UV/Vis spectrophotometer (SHIMADZU, Kyoto, Japan). Growth curves were formed through plotting log OD_620_ against the sampling time (h).

### 4.7. Integrity of Cell Membranes

The impact of *C. thouarsii* extract on cell membrane integrity of the tested isolates was inspected through monitoring the release of materials absorbing at 260 nm (A260) [16]. The tested isolates were grown in nutrient broth and the OD_630_ was adjusted to be 0.4. Then, 1 mL of each bacterial suspension was centrifuged for 10 min at 11,000× *g*, and the pellet was resuspended in a solution of 0.5% NaCl. The final suspension was adjusted to an absorbance of 0.7 at 420 nm. The release of materials absorbing at 260 nm from bacterial cells was tracked over time utilizing an 1800 UV/Vis spectrophotometer (SHIMADZU, Kyoto, Japan).

### 4.8. Inner Membrane Permeability Assay

It was investigated by measuring the release of the *β*-galactosidase enzyme from the cytoplasm of the tested isolates using ONPG as an enzyme substrate [17]. An overnight bacterial suspension grown in nutrient broth and supplemented with 2% lactose was centrifuged, and the pellet was washed and resuspended in a solution of 0.5% NaCl. Then, 150 µL of 34 mM ONPG solution was added to 1.6 mL of the bacterial suspension. The produced ONP was detected over time via monitoring the increase in the absorbance at 420 using an ELISA reader (Sunrise Tecan, Männedorf, Switzerland).

### 4.9. Outer Membrane Permeability Assay

It was determined according to the method previously described [18,33]. In brief, a stock solution of 5 mM NPN in ethanol was diluted using potassium phosphate buffer (PBS) (pH 7.5) to reach a concentration of 20 μM. The fluorescence of the samples was measured using a fluorescence spectrophotometer (SHIMADZU, Kyoto, Japan) at an excitation and emission wavelength of 340 and 420 nm, respectively.

### 4.10. Membrane Depolarization Assay

The tested isolates were harvested by centrifugation and resuspended in PBS; then, they were stained using 5 µg/mL DiBAC_4_(3) (Molecular Probe). The cell staining was analyzed using an FACS verse flow cytometer (BD Biosciences, New York, NY, USA) [17,19].

### 4.11. SEM Examination

The tested isolates were examined using an electron microscope as previously described [20] in the electron microscope unit, Tanta University, Egypt, using SEM (Jeol-1200 ECII) (Akashi Seisakusho, Japan).

### 4.12. Evaluation of Efflux Activity Using Cartwheel Method

Efflux of EtBr was examined using the cartwheel method [21]. Tryptic soy agar plates containing EtBr with concentrations ranging from 0 to 2.5 mg/L were set-up and well protected from light. The plates were then divided into sectors and the tested bacterial suspensions were swabbed onto the plates starting from the center toward the edges, using the reference strain as a negative control, and then incubated overnight at 37 °C. The plates were inspected using an 1800 UV-Vis transilluminator (SHIMADZU, Kyoto, Japan), and the lowest concentration of EtBr that produced fluorescence of the bacterial isolates was determined.

### 4.13. qRT-PCR

qRT-PCR was utilized to determine the relative expression of the efflux pump genes (*nor*E, *acr*B, *mdf*A, and *yih*V) using *gap*A as a housekeeping gene [34]. All the measurements were carried out in triplicate, and they were expressed as mean ± SD values. Total RNA was extracted by the Purelink^®^ RNA Mini Kit (Thermo SCIENTIFIC, Waltham, MA, USA), and 1 μL of RNA was used for the synthesis of cDNA by the power first-strand cDNA kit (iNtRON Biotechnology, Seongnam, Korea). The sequences of primers used in the amplification of the tested genes are shown in (Supplementary Materials, Table S1). The amplification was performed by a Rotor-Gene Q5 plex instrument (Qiagen, Hilden, Germany) using a Power SYBR^®^ Green master mix (Thermo SCIENTIFIC, Waltham, MA, USA). The threshold cycle method (2^−ΔΔCT^) was utilized [35] to analyze the changes in gene expression in each sample relative to the control (bacterial isolates before treatment with plant extract and its expression was set to 1).

### 4.14. General Instruments

A JEOL ECA-500 II NMR spectrometer was used to record NMR spectra at 500 MHz for ^1^H and 125 MHz for ^13^C. DMSO-*d*_6_ or CDCl_3_ were used to dissolve NMR samples. Chemical shifts δ were standardized to the solvent resonances. A Thermo Scientific ISQ Quantum Access MAX Triple Quadrupole system, Xcalibur 2.1 software, and USA Mass Spectrometer were used. Jasco′s FT/IR-6100 spectrophotometer was used to record IR spectra on KBr discs. The “Galen Kamp-type” melting point apparatus was used. Polarimeter models from Rudolph Research Analytical, USA, were used to detect optical rotation. Material for column chromatography: silica gel (Merck, 70–230 mesh), Sephadex LH-20 (Sigma–Aldrich Chemical Co. St. Louis, MO, USA), ODS (RPC18, Merck, Germany), silica gel F_254_ (Merck, 70–230 mesh). Camag UV lamps at 254 and 366 nm were used to observe the results. For spot detection, AlCl_3_ or 10% sulfuric acid spray reagents were used independently.

### 4.15. Statistical Analysis

The one-way analysis of variance test (ANOVA) was used to analyze the results via SPSS software. The results with *p* significance values of <0.05 were regarded as statistically significant.

## 5. Conclusions

*K. pneumoniae* pathogenic bacteria can cause many infections in addition to its multidrug resistance making its treatment more difficult. To overcome this problem, research on the anti-pathogenic and anti-infective effects of different plant extracts is increasing. In our study, *C. thouarsii* extract had antibacterial activity against *K. pneumoniae* clinical isolates. In addition, it inhibited bacterial growth and significantly decreased the membrane integrity of the bacterial cells. It also significantly decreased the inner and outer membrane permeability. Based on the scanning electron microscopy images, the extract had a drastic effect on the morphology of *K. pneumoniae* cells. The efflux inhibitory effect of *C. thouarsii* extract was investigated using the EtBr cartwheel method, and it was confirmed by examination of its influence on the expression of the genes encoding efflux pumps using qRT-PCR. A significant decrease in the expression of the efflux pump genes was detected in the selected isolates with percentages ranging from 33.3% to 55.5%. Further studies are highly needed soon to check the pharmacokinetic and pharmacodynamics properties of *C. thouarsii* extract to make use of its clinical value. One new biflavonoid 5,7,7”,4”′-tetra-*O*-methyl-hinokiflavone (**3**) was isolated from *C. thouarsii* leaves, in addition to five known compounds for the first time (stigmasterol, naringenin, 2,3-dihydrobilobetin, 4′,4‴-*O*-dimethyl amentoflavone or isoginkgetin, hinokiflavone). The MICs of the isolated compounds ranged from 0.25 to 2 µg/mL. Thus, *C. thouarsii* could be a promising source for new antimicrobials.

## Figures and Tables

**Table 1:** Efflux pump activity before and after treatment with *C. thouarsii* extract at different concentrations of EtBr using the cartwheel method.

EtBr Concentration (mg/L) *	Number of Bacterial Isolates (before Treatment)	Number of Bacterial Isolates (after Treatment)
≤0.5	10	23
1	11	4
1.5	9	6
2	4	1

* Concentration at which bacteria started to fluoresce considering the bacterial isolates lacking the efflux activity if it fluoresced at 0.5 mg/L and having an efflux activity if it fluoresced at higher concentrations.

**Table 2:** Relative gene expression (mean ± SD) for the tested *K. pneumoniae* isolates after treatment with *C. thouarsii* extract (2–16 µg/mL).

Isolate Code	Relative Gene Expression *			
	***nor*** **E**	***acr*** **B**	*mdf*A	*yih*V
K1	**0.1 ± 0.3**	1.2 ± 0.4	1.1 ± 0.3	0.3 ± 0.2
K2	**0.4 ± 0.2**	**0.3 ± 0.1**	**0.5 ± 0.2**	0.4 ± 0.3
K3	1.1 ± 0.1	1.5 ± 0.1	**0.6 ± 0.1**	1.5 ± 0.2
K4	1.2 ± 0.3	**0.5 ± 0.1**	**0.1 ± 0.1**	**0.6 ± 0.2**
K5	**0.5 ± 0.1**	**0.3 ± 0.2**	**0.3 ± 0.4**	1.1 ± 0.2
K6	1.3 ± 0.2	1.2 ± 0.1	1.2 ± 0.8	**1.6 ± 0.1**
K7	1.1 ± 0.3	**0.6 ± 0.0**	1.3 ± 0.2	**0.4 ± 0.2**
K8	1.4 ± 0.0	**0.2 ± 0.5**	1.4 ± 1.1	1.4 ± 0.3
K9	0.9 ± 0.2	1.4 ± 0.2	**0.3 ± 0.0**	**0.2 ± 0.2**

* The bolded values point to a significant decrease in gene expression (*p* < 0.05).

## Data Availability

The authors confirm that the data supporting this study are available within the article and its supplementary materials.

## Supplementary Materials

The following are available online at https://www.mdpi.com/article/10.3390/ph14080756/s1, Figure S1: IR spectrum of compound **3**; Figure S2: ^1^H-NMR spectrum of compound **3**; Figure S3: ^13^C-NMR spectrum of compound **3**; Figure S4: HMBC spectrum of compound **3**; Figure S5: COSY spectrum of compound **3**; Figure S6: ESI mass spectrum of compound **3**; Scheme S1: Column chromatography of methylene chloride fraction of *C. thouarsii* leaves.
