# Supplementary material for: Investigation of the Antibacterial Activity and Efflux Pump Inhibitory Effect of Cycas thouarsii R.Br. Extract against Klebsiella pneumoniae Clinical Isolates
Source: Pharmaceuticals (Basel). 2021 Aug 1;14(8):756. doi: 10.3390/ph14080756 (PMC8401967; doi:10.3390/ph14080756)
Supplement: Supplementary file 1 [file pharmaceuticals-14-00756-s001.zip › pharmaceuticals-1306360-supplementary.pdf]

<u>Supporting information</u>

Investigation of the antibacterial activity and efflux pump inhibitory effect of *Cycas thouarsii* R.Br. extract against *Klebsiella pneumoniae* clinical isolates.

Walaa Negm[1], Mona El-Asar[1], Amal Abokamer[2], Engy Elekhnawy[2]

[1] Pharmacognosy department, faculty of pharmacy, Tanta University, Tanta, El Gharbia governorate, Egypt.
[2] Pharmaceutical microbiology department, faculty of pharmacy, Tanta University, Tanta, El Gharbia governorate, Egypt.

Table S1: Sequences of oligonucleotides utilized in RT–PCR

**Tested genes**	**Primer***	**Sequence**
norE	F	5'-TCGCAGGACATCAGATTG-3`
	R	5'-CAGACACCCACCATAAGC-3`
*acr*B	F	5'-GAAGAGCACGCACCACTACAC-3`
	R	5'-GCAGACGCACGAACAGATAGG-3`
*mdf*A	F	5'-TTTATGCTTTCGGTATTGG-3`
	R	5'-GAGATTAAACAGTCCGTTGC-3`
*yih*V	F	5'- GGCTATCATCCTCGTCTTCC-3`
	R	5'- GCGTCATCCACCAGTAACC-3`
*gap*A	F	5'-GGACGAAGTTGGTGTTGAC-3`
	R	5'-TTCTGAGTAGCGGTAGTAGC-3`

*F: forward, R: reverse

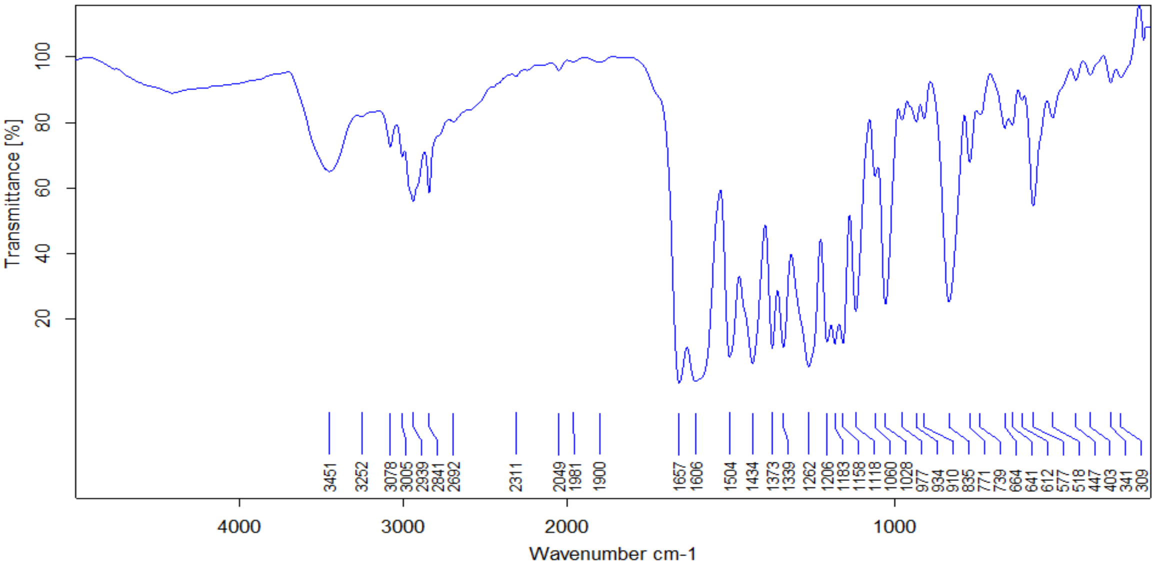

Figure S1. IR spectrum of compound **3**

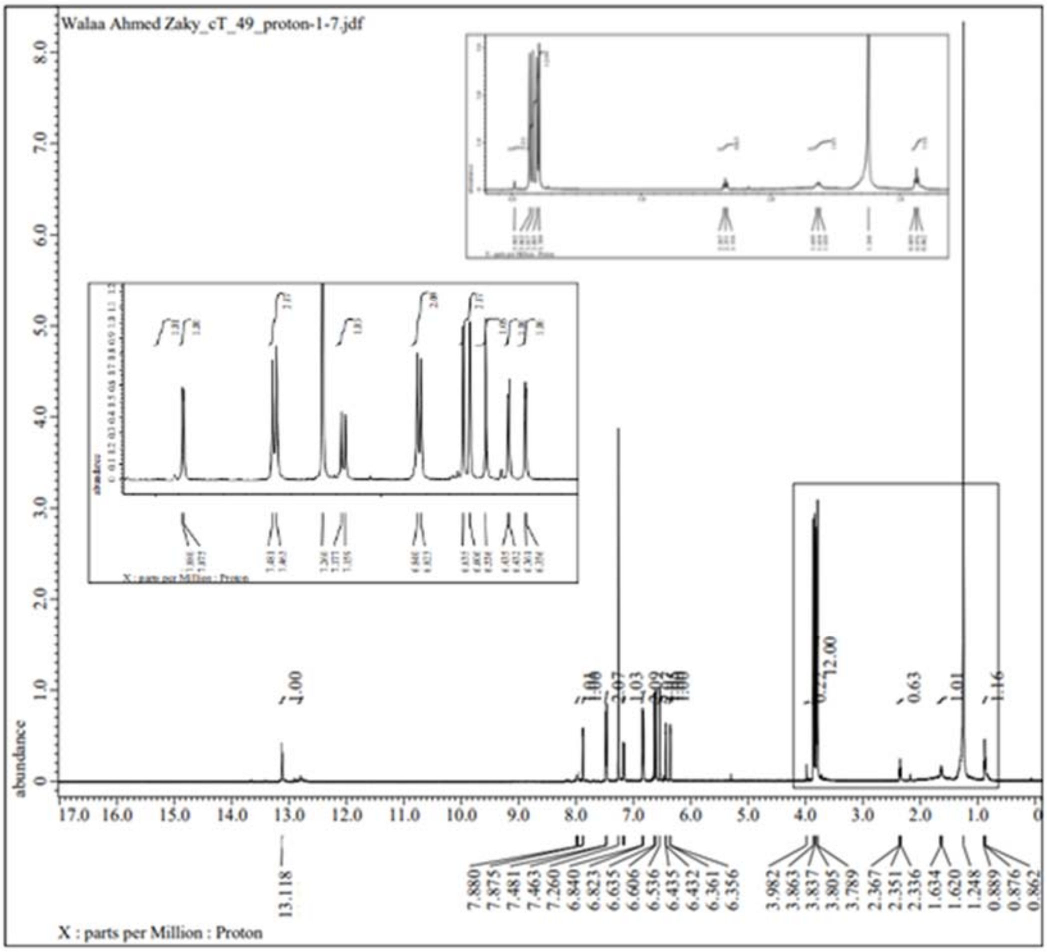

Figure S2. ^{1}H-NMR spectrum of compound **3**

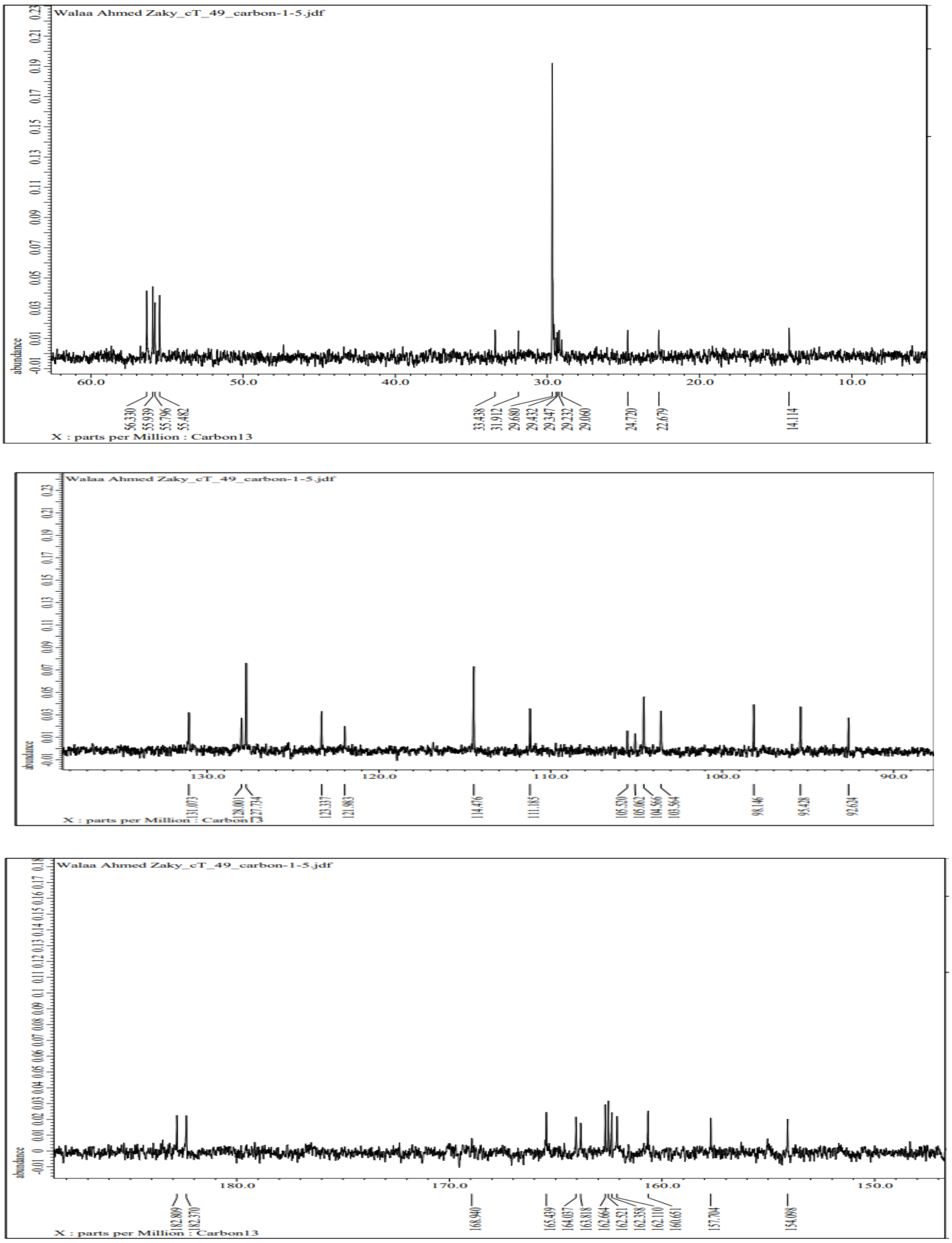

Figure S3. ^{13}C-NMR spectrum of compound **3**

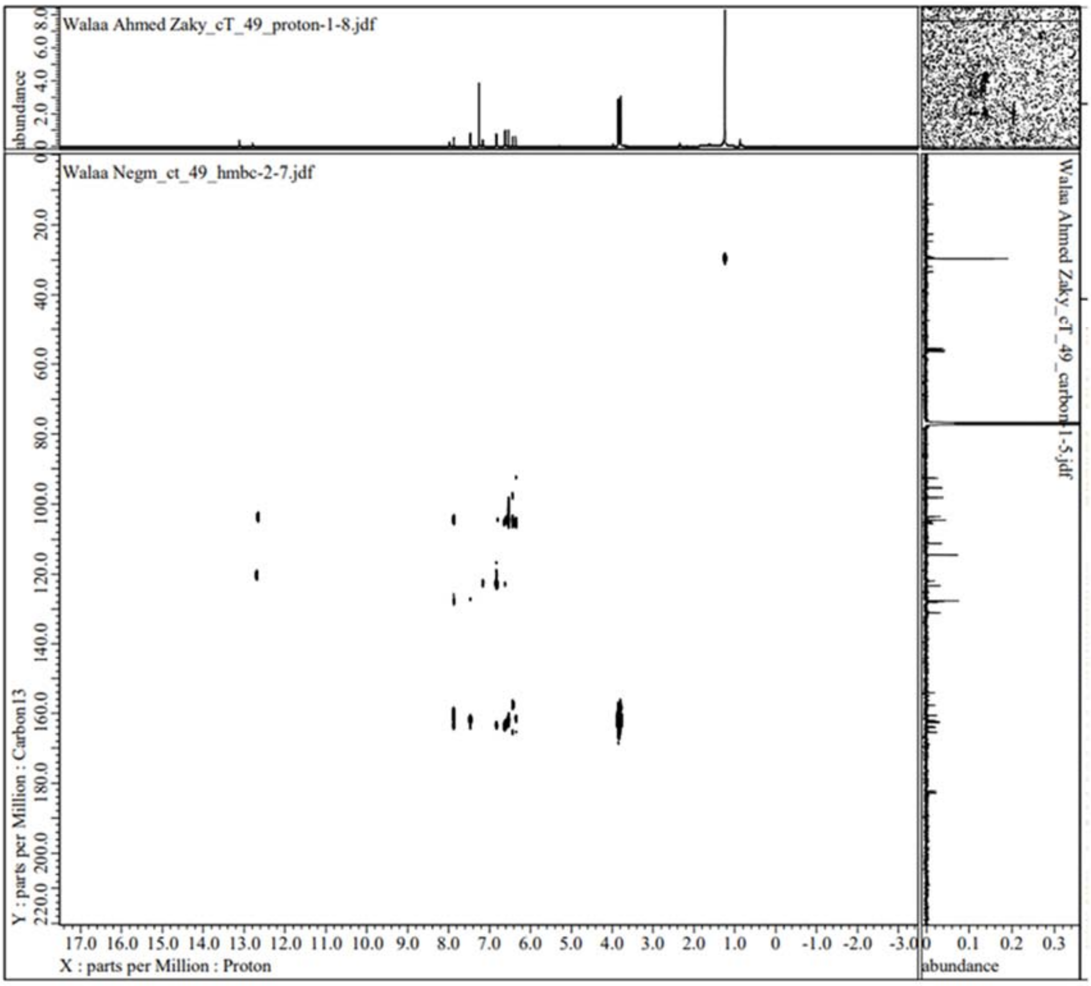

Figure S4. HMBC spectrum of compound **3**

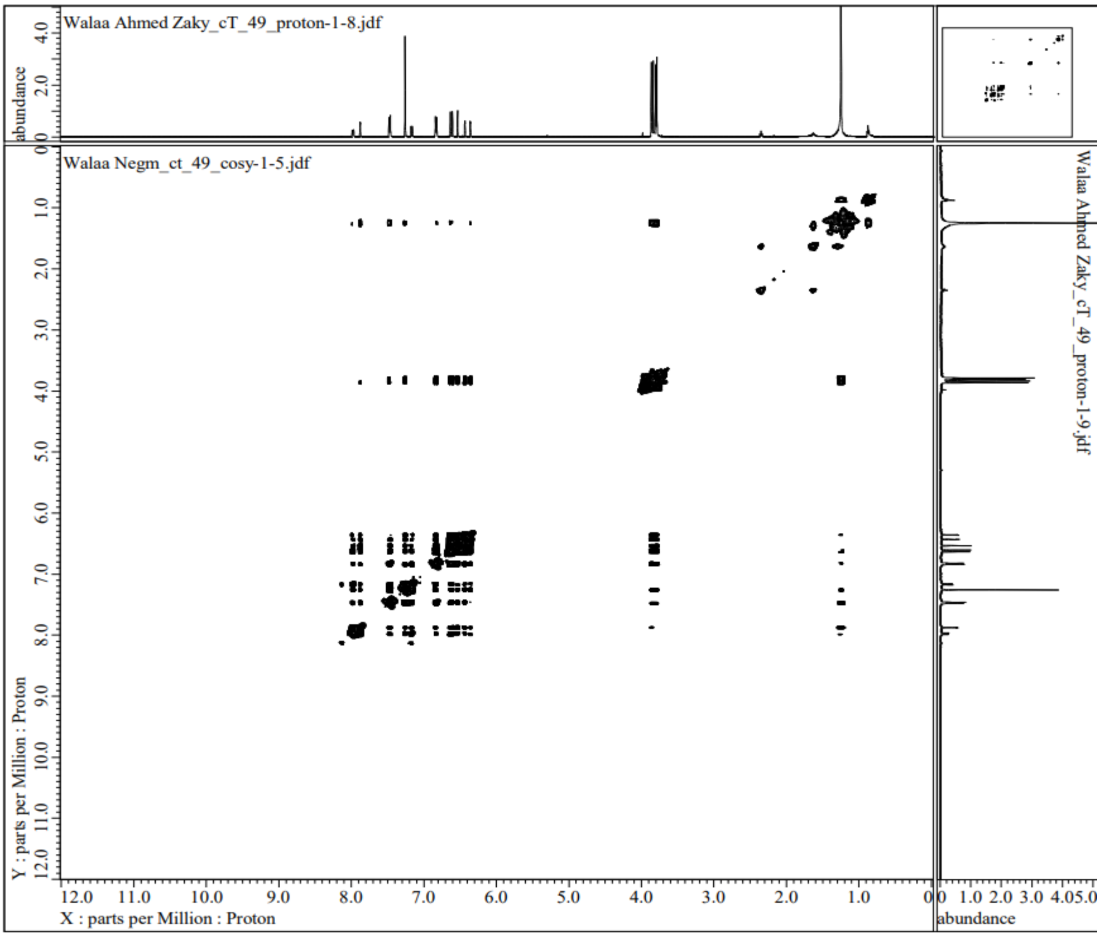

Figure S5. COSY spectrum of compound **3**

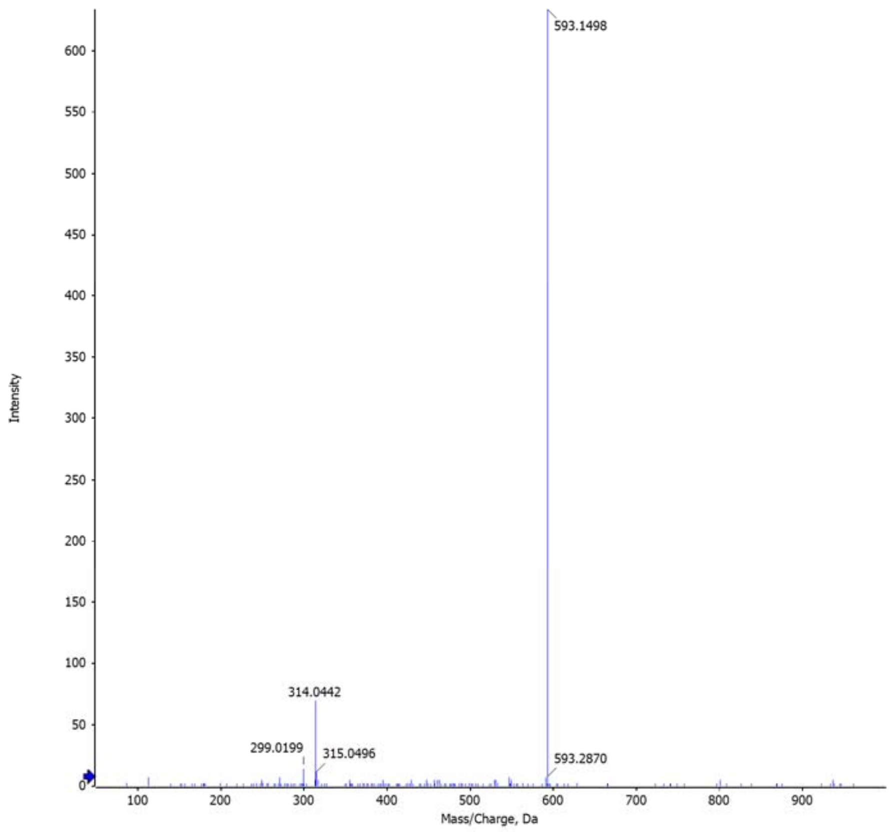

Figure S6. ESI mass spectrum of compound **3**

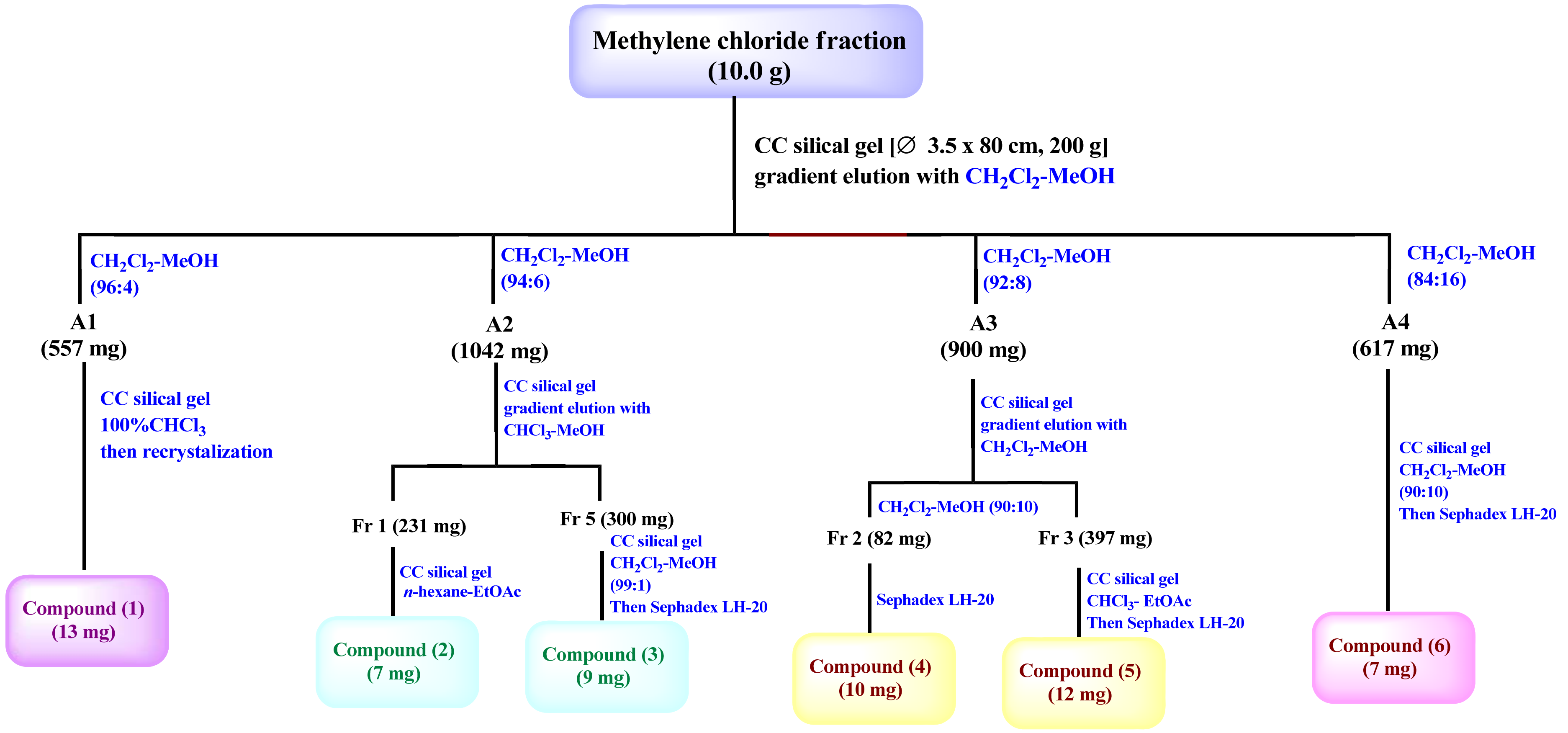

Scheme S1. Column chromatography of methylene chloride fraction of *Cycas thouarsii* leaves.
